# Changes in chlamydia control activities in Europe between 2007 and 2012: a cross-national survey

**DOI:** 10.1093/eurpub/ckv196

**Published:** 2015-10-24

**Authors:** Ingrid V. van den Broek, Otilia Sfetcu, Marianne A. van der Sande, Berit Andersen, Björn Herrmann, Helen Ward, Hannelore M. Götz, Anneli Uusküla, Sarah C. Woodhall, Shelagh M. Redmond, Andrew J. Amato-Gauci, Nicola Low, Jan E. van Bergen

**Affiliations:** ^1^Unit of Epidemiology and Surveillance, RIVM/Centre for Infectious Disease Control Netherlands, Bilthoven, The Netherlands; ^2^European Centre for Disease Prevention and Control (ECDC), Stockholm, Sweden; ^3^Julius Centre, UMC Utrecht, Utrecht, The Netherlands; ^4^Department of Public Health, Randers Hospital, Skovlyvej, Randers, Denmark; ^5^Section of Clinical Bacteriology, Department of Medical Sciences, Uppsala University, Uppsala, Sweden; ^6^Infectious Diseases Epidemiology, School of Public Health, Imperial College London, St Mary’s Campus, Norfolk Place, London, UK; ^7^Department of Infectious Disease Control, Municipal Public Health Service Rotterdam-Rijnmond, Rotterdam, The Netherlands; ^8^Department of Public Health, University of Tartu, Tartu, Estonia; ^9^HIV & STI Department, National Centre for Infectious Disease Surveillance and Control, Public Health England, London, UK; ^10^Institute of Social and Preventive Medicine, University of Bern, Bern, Switzerland; ^11^Department of General Practice, University of Amsterdam, Amsterdam, The Netherlands; ^12^STI AIDS Netherlands, Amsterdam, The Netherlands

## Abstract

**Background: **In 2012, the levels of chlamydia control activities including primary prevention, effective case management with partner management and surveillance were assessed in 2012 across countries in the European Union and European Economic Area (EU/EEA), on initiative of the European Centre for Disease Control (ECDC) survey, and the findings were compared with those from a similar survey in 2007. **Methods: **Experts in the 30 EU/EEA countries were invited to respond to an online questionnaire; 28 countries responded, of which 25 participated in both the 2007 and 2012 surveys. Analyses focused on 13 indicators of chlamydia prevention and control activities; countries were assigned to one of five categories of chlamydia control. **Results: **In 2012, more countries than in 2007 reported availability of national chlamydia case management guidelines (80% vs*. *68%), opportunistic chlamydia testing (68% vs*. *44%) and consistent use of nucleic acid amplification tests (64% vs*. *36%). The number of countries reporting having a national sexually transmitted infection control strategy or a surveillance system for chlamydia did not change notably. In 2012, most countries (18/25, 72%) had implemented primary prevention activities and case management guidelines addressing partner management, compared with 44% (11/25) of countries in 2007. **Conclusion:** Overall, chlamydia control activities in EU/EEA countries strengthened between 2007 and 2012. Several countries still need to develop essential chlamydia control activities, whereas others may strengthen implementation and monitoring of existing activities.

## Introduction

Chlamydia is the most commonly reported infection in the European Union and the European Economic Area (EU/EEA).^1^ Untreated, chlamydia infections can cause complications, including pelvic inflammatory disease, which may lead to ectopic pregnancy or tubal factor infertility in women, and epididymitis in men.^2^^,^^3^ The implementation of chlamydia control activities influences who is tested and how many cases are detected and reported.^4^ Surveillance data from EU/EEA Member States, collected in The European Surveillance System (TESSy, ECDC), show that the number of cases reported has increased over time and varies widely between countries (from <1 to 600 per 100 000 population in 2012).^5^ Countries across Europe differ in the priority they give to the control of sexually transmitted infections (STI) such as *Chlamydia trachomatis* (chlamydia) and in their level of implementation of primary prevention, STI services and surveillance data collection.^6^^,^^7^

The ECDC first assessed activities aimed at the control of chlamydia infections in EU/EEA Member States in 2007.^6^^,^^7^ Survey responses were used to define categories of chlamydia control, according to the level of infrastructure required to implement the activities, and to allocate each country to a category. ECDC used the survey findings to publish a guidance document in 2009 that aimed to support countries to strengthen their national control strategies. The guidance document suggested a step-by-step approach to chlamydia control,^8^ with essential activities, policies and evaluation indicators.

In 2012, ECDC conducted a second survey of chlamydia control activities in EU/EAA Member States. The objectives were: to assess the organization and implementation of chlamydia control activities, including primary prevention; and to examine changes between 2007 and 2012.

## Methods

The survey methods are described in full in a technical report.^9^ ECDC invited national STI surveillance contacts from EU/EEA Member States to respond to an online questionnaire between December 2012 and February 2013. The questionnaire included 63 questions in six sections: (i) guidelines on chlamydia case management and testing; (ii) laboratory diagnosis; (iii) strategies, plans and organization of STI healthcare; (iv) strategies and activities for primary prevention; (v) surveillance and (vi) opportunistic testing and screening programmes.^9^ The 2012 questionnaire was adapted to use an online survey tool (Sharepoint, Microsoft); most questions were similar to the 2007 survey (Microsoft Word, sent electronically). Questions about primary prevention were new. Respondents were also invited to indicate the level of chlamydia control that described the situation in their country, based on the levels described in the ECDC guidance (A to D, explained below) and comment on chlamydia control activities. The survey included questions to evaluate the use of the guidance document which have been analysed and will be reported separately.^10^

The survey team developed a set of 13 ‘key indicators’ of chlamydia prevention and control activities (Supplementary table S1), based on the questionnaire sections and the ECDC guidance document.^8^ Data for 2012 were collated and merged with responses from 2007. The findings per key indicator were described for 2012 and responses compared between 2007 and 2012 for questions asked and for countries taking part in both surveys.

Survey responses in 2012 were then used to determine a country’s category of control activities. Two investigators independently assigned each Member State to a category, using the same method as in the 2007 survey.^7^ In cases of discordant classification the investigators reached a consensus by discussion or, if necessary, a third reviewer adjudicated. The five categories are shown in table 1. The numbers of countries in each category in 2007 and 2012 were compared.

**Table 1:** Chlamydia control activities of EU/EEA Member States: control category, based on country responses in the ECDC survey in 2012 using the criteria described in column 2 and the corresponding level of control activities (A–D) in accordance with the ECDC guidance 2009 (column 3) and levels based on the country’s self-assessment

Chlamydia control category 1–5	Criteria	ECDC guidance (2009) activities level	Countries per category (with self-assessed level^a^)	*N* (%)
**No organized **chlamydia**control activities**	No national guidelines for chlamydia diagnosis and treatment		**Malta (C), Portugal (A), Slovakia (A), Slovenia (A), Ireland (n.a.), Luxembourg (n.a.)**	6 (21%)
**Case management for diagnosed **chlamydia**cases (case management)**	Guidelines on chlamydia diagnosis and treatment for at least one group of healthcare professionals	B	**Belgium (B), Cyprus (A), Italy (B)**	3 (11%)
**Case finding for partners of diagnosed **chlamydia**cases (case management, including partner notification)**	Case management guidelines plus partner notification	B	**Czech Republic (B), Hungary (A), Liechtenstein (B), Romania (B), Spain (B)**	5 (18%)
**Opportunistic testing for selected asymptomatic individuals (opportunistic testing)**	Case finding plus chlamydia testing offered to at least one specified group of asymptomatic people	C	**Austria (B), Denmark (B), Estonia (B), Finland (C), France (C), Germany (D), Iceland (C), Lithuania (A), Netherlands (C), Norway (C), Sweden (C), Bulgaria (n.a.), Latvia (n.a.)**	13 (46%)
**Organized screening programme (screening programme)**	Organized chlamydia screening available to a substantial part of the population within the public health system with defined organizational characteristics	D	**UK (D)**	1 (4%)

n.a.= not applicable: country representative(s) indicated ‘does not fit any category’ (Ireland, Bulgaria, Latvia) or did not answer this question (Luxembourg). UK situation based on England.

^a^Self-assessed levels used the scale of the ECDC guidance level A–D^8^ while categories 1–5 were used in both ECDC surveys in 2007 and 2012.^7,9^

The assigned category (1–5) for each country in 2012 was mapped to the respondent’s self-assessed level (A to D) because future assessments of chlamydia prevention and control activities will be based on criteria in published ECDC guidance. The survey categories and levels in the 2009 guidance partly overlap, as shown in table 1. Level A describes activities for the primary prevention of STI but no other organized chlamydia control activities; category 1 does not mention prevention because the 2007 survey did not include this topic; level B covers categories 2 and 3, requiring clinical chlamydia guidelines either or not include partner notification; level C and D and category 4 and 5 are the same.

Secondary data [population size, per capita gross domestic product (GDP)^11^] and rates of chlamydia cases reported to ECDC in 2011^5^ were also recorded. Information about the completeness of chlamydia surveillance (this survey and ECDC report^5^) was used to estimate the number of cases per 100 000 for each country with complete reporting. These estimated rates were compared to the assigned category of control activities. All analyses were done with SPSS Statistics (version 19.0, IBM, New York).

## Results

Twenty-eight of 30 (93%) EU/EEA Member States responded to the survey in 2012; Greece and Poland did not respond. Luxembourg reported that chlamydia control had not changed, so responses from 2007 were used. For 25 countries data were available from both surveys (Slovakia, Cyprus, Czech Republic had no data for 2007).

### National strategies for sexual health promotion and STI control (key indicator 1–3)

**Figure 1:**
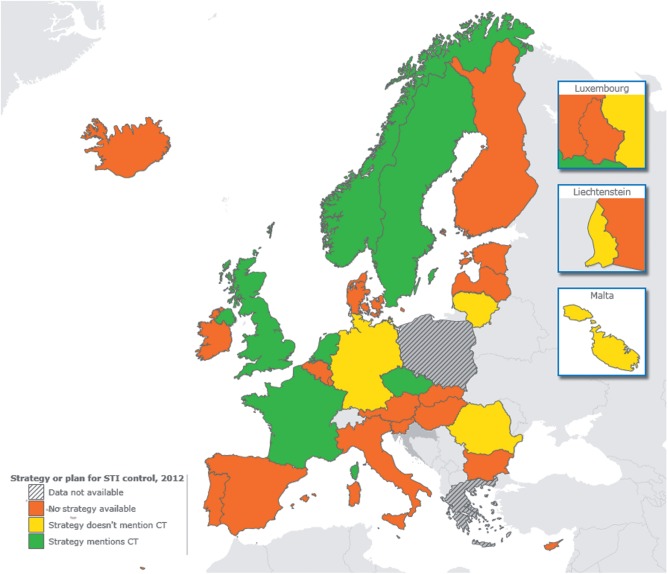
EU/EEA countries with a national strategy or plan about STI control in 2012. CT = chlamydia. UK situation based on England. Adapted from ECDC Report^7^

A total of 16/27 (59%) countries reported a national sexual health promotion strategy in 2012 (no information available from Luxembourg). Most countries (22/27; 81%) reported at least one organized activity to improve knowledge, behaviour or awareness of chlamydia including regular media campaigns (6/22), social media campaigns (13/22), sexual health education as standard (11/22) or voluntary (7/22) part of school curricula. Eleven of 28 countries (39%) reported a national strategy/plan for STI control; six explicitly included chlamydia control (figure 1). Among countries participating in both surveys, 8/25 (32%) reported national STI control strategies in 2007 compared with 10/25 (40%) in 2012. In addition, Sweden and Denmark revised their strategies; both reported a shift in priorities towards primary prevention of STI.

### Health services for STI care (key indicator 8)

Dedicated health services offering diagnosis and treatment for people with STI were available in 25/28 (89%) countries in specialized or general healthcare facilities. Liechtenstein, Luxembourg and Slovakia reported no public dedicated STI services. Availability of services for STI care was similar in 2007 (22/25 countries; 88%) and 2012 (23/25; 92%). Listed among the three most common providers of STI care per country were: gynaecologists (25/28, 89%), STI/GUM/dermatovenereology clinics (23/25, 82%), urologists (18/28, 64%), family planning or contraception clinics (14/28, 50%) and general practitioners (12/28, 43%). Belgium included hospital services for infectious diseases and Sweden mentioned a medical service for ordering homesampling tests via the Internet as one of the top three providers.

In 11 of 28 countries (39%), the costs of consultation, diagnosis, treatment and partner notification within STI care were fully covered or reimbursed by national health insurance; in 5 countries (18%) costs were partially covered, in 10 countries (36%) only some were (partially) covered, whereas in 2 countries none was covered. Costs of consultation and diagnosis were more often covered than costs of treatment or partner notification. In 2012, costs were completely covered in 10/25 countries (40%) compared with 8/25 (32%) in 2007.

### Diagnostic tests for C. trachomatis detection (key indicator 9–10)

Nucleic acid amplification tests (NAAT) to detect *C. trachomatis* were available in all countries that participated in 2012. In 22 countries NAAT was the most common diagnostic method in the public sector; in five countries other diagnostic methods were still more common: direct immunofluorescence microscopy (Cyprus, Hungary, Romania), enzyme-linked immunosorbent assay (Bulgaria, Portugal) and chlamydia culture (Romania). Seventeen of 28 countries (61%) reported that NAAT was used for >90% of samples tested. In 2007, 9 (36%) and in 2012, 16 (64%) of 25 countries taking part in both surveys used NAAT i*n* >90% of tests.

Questions on further testing capacity for chlamydia were answered by 22 countries: 18 (82%) had laboratory capacity to detect *C. trachomatis* variants that might not be detected by routine NAATs (such as the new variant described in Sweden^12^), 19 (86%) had capacity to detect L-genotypes causing *Lymphogranuloma venereum* and eight countries could assess *C. trachomatis* antimicrobial susceptibility.

### Case management guidelines (key indicator 4, 6)

In 2012, 22/28 (79%) countries had at least one national guideline that covered chlamydia diagnosis and treatment for one or more medical professions; in total 68 guidelines were reported. Nineteen countries (68%) had guidelines for all healthcare practitioners, all with additional specialist guidelines except Estonia. In 17 countries one or more guidelines were new or updated since the last survey. There were more guidelines in 2012 (62 guidelines in 20/25 countries; 80%) than in 2007 (25 guidelines in 17/25; 68%).

#### Partner notification

Case management guidelines explicitly addressed case finding through partner notification in 19/28 (68%) countries in 2012. In nine countries guidelines recommended patient referral; in seven countries, recommended partner notification practices included (also) provider referral (five countries), patient delivered partner therapy (three countries) or a choice between patient/provider referral depending on the situation (one country).

#### *Opportunistic testing for *chlamydia

In 18/28 (64%) countries, case management guidelines recommended opportunistic chlamydia testing for one or more specific groups of asymptomatic people; three country respondents stated that opportunistic testing was implemented routinely; 12 indicated limited/infrequent implementation and three reported recommendations were not implemented. Target groups for testing included young people (10/18; when specified this was <25 years except in France where the age group was 15–30 years), pregnant women (10/18) and other high risk groups, such as those practising sex without a condom (1/18), after partner change (3/18) or men who have sex with men (6/18). Respondents also specified clinical indications: notified partners or before gynaecological procedures. In 2012, the number of countries with opportunistic testing in guideline(s) (17/25; 68%) was higher than in 2007 (11/25; 44%).

#### Repeated testing

Case management guidelines recommended repeated testing after positive chlamydia tests in 14 countries: to confirm a positive test (two countries); as a test of cure, typically 3–6 weeks after diagnosis, for all cases (six countries) or specific cases (four countries) such as suspected poor compliance, persistent symptoms or non-standard treatment; or testing for reinfection, several months after infection (seven countries). Annual repeated screening was recommended in four countries.

### Chlamydiascreening programmes (key indicator 7)

For this survey, a chlamydia screening programme was defined as a continuing organized service that screens a high enough proportion of the target population at regular intervals to achieve defined targets, while minimizing harm.^9^^,^^13^ One country (England, UK) reported a screening programme based on opportunistic testing. The Netherlands stopped a pilot of a population-based screening programme in 2012. Germany reported reimbursement of chlamydia screening tests for young women since 2008, which fulfils the national definition of a screening programme; this activity corresponds to our definition of opportunistic testing. In 2012, fewer countries had (plans for) organized chlamydia screening programmes (4/25, 16%) than in 2007 (11/25; 44%). Of the three countries with plans in 2012, France and Luxembourg also reported this intention in 2007, whereas Malta’s plan was new. There were plans to introduce or pilot an organized chlamydia screening programme in three other countries in 2007, but these were not reported in 2012.

### Surveillance (key indicator 11–13)

A national surveillance system to report and monitor diagnosed chlamydia infections was in place in 25/28 (89%) countries. Austria and Portugal reported no national chlamydia surveillance system and Spain informed about plans to introduce a system in 2013. In 19/25 (76%) countries with a surveillance system, reporting from all settings was compulsory (comprehensive mandatory reporting, table 2). In six countries, reporting was done in selected sentinel settings. The reported types of surveillance systems were similar in 2007 (22/25; 88%) and 2012 (23/25; 92%).

**Table 2:** Category of chlamydia control, type of surveillance systems and reporting rates in EU/EEA Member States, ranked by the estimated number of cases per 100 000 population (last column)

Country	Category of chlamydia control 2012	Type of surveillance system as reported in questionnaire	Number of cases reported to ECDC in 2011^5^	Cases per 100,000 in 2011^5^	Estimated^a^ coverage: % of diagnosed cases reported in surveillance	Estimated number of cases per 100 000^b^
**Iceland**	4	Comprehensive, mandatory	2091	657	Complete	657
**Denmark**	4	Comprehensive, mandatory	26 617	479	Complete	479
**Norway**	4	Comprehensive, mandatory	22 530	458	Complete	458
**Sweden**	4	Comprehensive, mandatory	37 209	396	Complete	396
**UK**	5	Comprehensive, mandatory	213 398	341	Complete	341
**Finland**	4	Comprehensive, mandatory	13 667	254	Complete	254
**Netherlands**	4	Sentinel system (high risk; specific criteria)	12 926	—	35%	222
**Estonia**	4	Comprehensive, mandatory	1763	128	60–70%	199
**Ireland**	1	Comprehensive, mandatory	6407	143	Complete	143
**Latvia**	4	Comprehensive, mandatory	1576	70	Underreporting	>70
**Liechtenstein**	3	Comprehensive, mandatory	—	—	Unknown	41^c^
**Belgium**	2	Sentinel system, laboratory reporting, voluntary	3566	—	70%	46
**Malta**	1	Comprehensive, mandatory	155	35	Underreporting	>35
**Italy**	2	Sentinel system	339	—	4%	14
**Austria**	4	No surveillance system; ‘sentinel’ reporting, voluntary (high risk; CSW)	1004	—	Unknown	12
**Slovenia**	1	Comprehensive, mandatory	232	11	Underreporting	>11
**Lithuania**	4	Comprehensive, mandatory	343	11	Complete	11
**Slovakia**	1	Sentinel system ^d^	306	6	60%	9
**Hungary**	3	Comprehensive, mandatory ^d^	858	—	Unknown	9
**Spain**	3	No surveillance system; laboratory reporting, voluntary^e^	905	—	25%	8
**Cyprus**	2	Comprehensive, mandatory	6	0.7	Underreporting	>0.7
**Bulgaria**	4	Comprehensive, mandatory	55	0.7	Complete	0.7
**Romania**	3	Comprehensive, mandatory	133	0.6	underreporting	>0.6
**Luxembourg**	1	Comprehensive, mandatory	1	0.2	Unknown	0.2
**Portugal**	1	No surveillance system	—	—	—	Unknown
**Czech Republic**	3	Comprehensive, mandatory (since 2012)	—	—	Unknown	Unknown
**France**	4	Sentinel system, laboratory reporting, voluntary	—	—	Unknown	Unknown
**Germany**	4	Sentinel system	—	—	Unknown	Unknown

^a^Based on country responses in the survey questionnaire; UK situation based on England.

^b^The last column, ‘Estimated cases per 100 000’ is based on the number of cases reported to ECDC 2011,^5^ with a correction for coverage rate applied if the country’s STI expert answering the survey provided an estimate of the proportion of cares coverage by the surveillance system (second to last column).

^c^Liechtenstein reported 15 chlamydia cases in 2012 in the survey questionnaire, corresponding to 41/100 000 reported cases.

^d^Slovakia has comprehensive surveillance and Hungary has sentinel surveillance, according to the ECDC surveillance report.^5^

^e^Spain is in the process of implementing a new surveillance protocol for comprehensive, mandatory reporting.

Nine of the 25 countries (36%) indicated their data collection was complete and covered (nearly) all cases detected annually (table 2). Reasons for partial coverage were: organizational, i.e. sentinel surveillance; voluntary laboratory reporting and under-reporting by STI health care providers. Under-reporting of cases was the main reason given by countries with low rates of reported chlamydia. Surveillance data included numbers of chlamydia tests in 9/25 (36%) countries.

Ten of 28 countries collected data on clinical complications caused by chlamydia. Seven countries collected data on Pelvic Inflammatory Disease (PID), five on ectopic pregnancy, three on infertility and two on epididymitis.

### Level of chlamydia control activities

**Figure 2:**
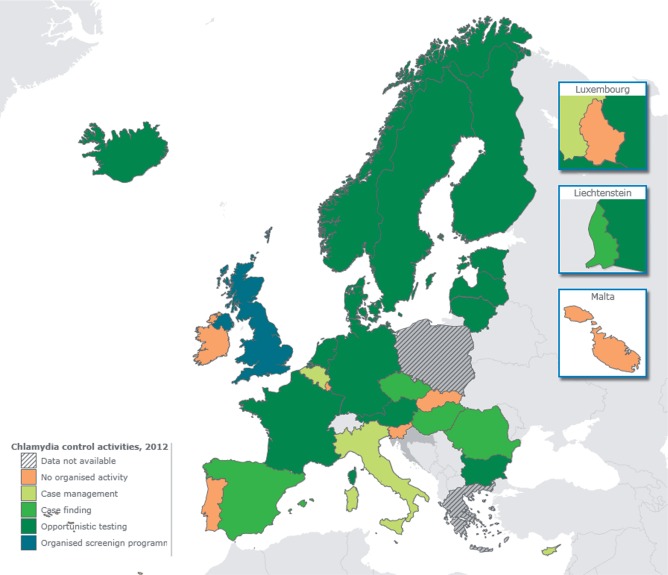
Map of Europe indicating the level of chlamydia control based on the countries’ accomplishments of key indicators assessed in the survey in 2012. UK situation based on England. Adapted from ECDC Report^7^

Nineteen of 28 (68%) countries had activities in categories 3–5 (table 1 and figure 2), an increase since the previous survey: for the 25 countries participating in both surveys, 18 (72%) in 2012 and 11 (44%) in 2007 were in categories 3–5. Twenty-seven countries completed the self-assessment of the level of chlamydia control. Three countries (3/27; 15%) reported that they did not think they fitted into any of the levels described. The remainder classified their country as level A, 6/27 (22%); level B, 9/27 (33%); level C, 7/27 (26%); level D, 2/27 (7%). Comparing the self-assessment to the categories assigned by the survey team (table 1), six countries assigned themselves to a level with less intensive activities than assigned by the survey team, while four countries selected a level with more intensive activities.

There was no statistical evidence for a difference between mean GDP per capita in 2012 of countries classified in five categories or regrouped in two categories (category 1 and 2 vs. category 3 or higher). The assigned category of chlamydia control was not clearly related to the type of surveillance system, but appeared to be related to the estimated reporting rate (table 2). Seven countries (all in categories 4 or 5, Denmark, Finland, Iceland, Netherlands, Norway, Sweden and UK) reported more than 90% of chlamydia cases recorded by ECDC in 2011, but comprise only 23% of the EU/EEA population. These countries implement recommendations for opportunistic chlamydia testing and (except Netherlands) surveillance systems with comprehensive coverage. Five countries, Czech Republic, France, Germany, Liechtenstein and Portugal, (37% EU/EEA population) did not contribute data to ECDC case reports in 2011. Case reporting rates were low in most Eastern (e.g. Bulgaria, Hungary, Romania) and southern (Cyprus, Italy, Malta and Spain) European countries despite mandatory reporting systems in most of them; their level of chlamydia control ranged widely.

## Discussion

A comparison of two cross-sectional surveys found that, in 2012 more EU/EEA Member States than in 2007 reported organized chlamydia control activities. The number of countries with national chlamydia case management guidelines also increased. Compared with 2007, the availability of dedicated health services for STI care was generally maintained. More than half of EU/EEA Member States reported a national sexual health promotion strategy in 2012 but most had no documented STI control strategy. A significant progress was the increase in the consistent use of NAAT indicating increased accuracy of laboratory diagnoses; on the other hand, only four countries insured that all laboratories participated in an international quality assurance scheme. The surveillance of diagnosed cases has not changed since 2007.

The 2012 survey of chlamydia control had a high response rate that allowed a good comparison with the 2007 survey. There were two main limitations to the survey format. First, the questionnaire covered a variety of different specialist topics and one person often responded to all questions. Respondents might have misunderstood or misinterpreted some questions, although they were encouraged to consult others and could refer to a glossary of definitions. In addition, a single response for a whole country might not describe regional variations accurately, particularly in larger countries or where healthcare services are devolved. Second, the change of survey format between 2007 and 2012 and re-wording of some questions might have affected their comparability. To address this problem, we asked respondents for clarification if responses to the two surveys were contradictory.

EU/EEA Member States with more intense chlamydia control activities and more complete reporting to surveillance systems reported higher rates of diagnosed cases to TESSy.^5^ This finding, across a large geographic region, supports ecological studies in single countries that show that high rates of chlamydia cases reported in surveillance systems reflect more the coverage of testing^14^ and not the comparative incidence or prevalence of chlamydia. Estimates of chlamydia prevalence in countries with nationally representative surveys^3^^,^^15^ show similar conclusions, and suggest that many asymptomatic chlamydia infections are neither diagnosed nor reported in countries with low case reporting rates.

The number of EU/EEA Member States with national guidelines for chlamydia case management that include partner notification, increased between 2007 and 2012. However, the existence of recommendations does not necessarily mean that they are implemented; indeed STI experts in several countries remarked that they were unsure about the level and consistency of implementation. In 2012, more EU/EEA countries than in 2007 reported that guidelines recommend opportunistic testing, but only three indicated that it was implemented in routine practice.

Future assessments of chlamydia prevention and control can use the self-assessed levels (A to D) developed for the 2009 ECDC Guidance document on chlamydia control. The level for each country has been mapped to the previously assigned categories; discrepancies were minor and reflected the difference between what is described in guidelines and respondents’ statements about actual practices.

Reported chlamydia screening policies changed between 2007 and 2012. The fall in the proportion of countries with an ongoing or planned chlamydia screening programme could partly reflect uncertainty about the balance between benefits and harms of organized screening programmes.^13^ This change may also reflect changes in economic circumstances across Europe during the period between the surveys. In the Netherlands, the pilot programme of register-based chlamydia screening (reported in 2007) was discontinued in 2012 after a controlled trial showed limited uptake and no change in chlamydia test positivity after three rounds of screening.^16^ In England, opportunistic chlamydia screening has continued.^17^ Population-based estimates of chlamydia prevalence in 1999–2000 (before the programme started) and 2010–2012 were similar, however.^18^ No other European countries have done analyses showing if screening would be effective, except in Ireland where a modelling study concluded that an opportunistic chlamydia screening programme would be expensive to implement nationally and is unlikely to be judged cost-effective by policy makers.^19^ More insight in trends and determinants of chlamydia-associated complications is necessary. Sweden and Denmark, which have widespread opportunistic chlamydia screening, but no organized programme, reported that their STI control strategies had shifted from promoting testing to intensifying primary prevention activities.

The findings of this survey have implications for policy making, service provision and evaluation of chlamydia prevention and control. Most EU/EEA Member States still did not have a written STI control strategy in 2012. Despite the presence of dedicated services for STI control in most countries, treatment for chlamydia is provided free in less than half. Given the equipoise about the efficacy of organized chlamydia screening and the complex infrastructure required for its implementation, efforts should focus on ensuring implementation of the essential elements of STI control; primary prevention, comprehensive case management and surveillance. The 2012 survey identified a number of countries that still need to develop national sexual health promotion strategies and case management guidelines including partner notification. For countries already reporting these achievements, the next steps will be to improve their implementation and monitor the quality of care, so that recommendations on paper are aligned with daily practice. Overall, reported activities for chlamydia prevention and control in Europe strengthened between 2007 and 2012.

## Supplementary data

Supplementary data are available at *EURPUB *online.
